# Simple Detection Methods for Antinutritive Factor β-ODAP Present in *Lathyrus sativus* L. by High Pressure Liquid Chromatography and Thin Layer Chromatography

**DOI:** 10.1371/journal.pone.0140649

**Published:** 2015-11-02

**Authors:** Bidisha Ghosh, Joy Mitra, Saikat Chakraborty, Jagannath Bhattacharyya, Anirban Chakraborty, Soumitra Kumar Sen, Muniasamy Neerathilingam

**Affiliations:** 1 Protein Technology Core, Centre for Cellular and Molecular Platforms, NCBS-TIFR, Bangalore, Karnataka, India; 2 Advanced Laboratory for Plant Genetic Engineering, Indian Institute of Technology Kharagpur, Kharagpur, West Bengal, India; National University of Ireland - Galway, IRELAND

## Abstract

*Lathyrus sativus* L. *(Grass pea)* is the source for cheap and nutritious food choice in drought and famine susceptible zones in greater part of North India and Africa. The non-protein amino acid β-*N*-oxalyl-L-α,β-diaminopropionic acid (β-ODAP) has been known for decades for its potent neurotoxic effect, causing irreversible neurodegenerative disease “neurolathyrism”, present in both seed and leaf of *Lathyrus sativus* L. and other species in varying proportions. It is crucial to establish a rapid as well as reliable detection methodology for β-ODAP content in various Lathyrus plants. Currently available HPLC based methods involve multi-step derivatization of the sample. To overcome this, we have developed β-ODAP analysis method by HPLC without any prior derivatization. This method is statistically significant in the range of 2 to 100μg/ml and exhibited linear response with *r* ^*2*^ > 0.99. Limit of detection and quantitation of the later method was determined to be 5.56 μg/ml and 16.86 μg/ml, respectively. In addition to this, a TLC based method has also been developed. The limit of detection of β-ODAP is 0.6μg and for its substrate, L-1,2-diaminopropionic acid is 5μg. Both HPLC and TLC methods were validated by conducting *in-vitro* bioconversion test to detect the presence of biocatalyst in plant extract. This method is economical, rapid and simple.

## Introduction

Grass pea, *Lathyrus sativus* L. a versatile annual legume crop with natural ability to grow in waterlogged, high saline soil and drought prone areas [1]. It is a sustainable crop for long term cultivation by virtue of its nitrogen fixing ability, which enables it to grow in poor soil without application of fertilizer and capability to resist the pest and infections [2,3]. It is widely consumed as food in parts of Africa eg, Ethiopia and Asia eg, India, Bangladesh and Pakistan, Middle-East, Afghanistan and northwest China and Russia. Cultivation of this plant has been restricted globally because of its toxic reputation due to the presence of the neurotoxin β-*N*-oxalyl-L-α,β-diaminopropionic acid (β-ODAP) in its tissues. On prolonged consumption i.e. more than three months as main diet it may result in outbreak of the disease called “neurolathyrism”. This has been reported during 18th, 19th and 20th centuries throughout the countries where it was consumed regularly. It results in irreversible paralysis of the lower limb muscles in humans ensuing in loss of walking ability [4–7]. Other sporadic neurological signs have also been reported like cranial nerves as well as urinary bladder involvement and polyneuropathy [8]. Poverty, illiteracy and stress are risk factors while consumption of cereals and antioxidant containing condiments are protective factors in the epidemiology of neurolathyrism [9].

Grass pea is reported to contain 0.5–2.5% of β-ODAP [1]. Toxic β-ODAP is present in the seeds of 21 Lathyrus species (mainly *Lathyrus sativus* L., *Lathyrus cicera* L. and *Lathyrus*. *clymenum* L.) and some other genera of leguminous plants: 17 species of Acacia and 13 species of Crotalaria [10]. It is also present in some non-legume plants like ginseng roots of *Panax ginseng*, *P*. *notoginseng* and *P*. *quinquefolius* [11,12]. Effort is being made to breed genetic varieties of *Lathyrus sativus* L. which are safe for human and cattle consumption [13]. There are several analytical techniques for determination of β-ODAP content of grass pea. The different methods used are HPLC with derivatization, colorimetric assay, flow injection analysis, capillary zone electrophoresis, and near infrared reflectance spectroscopy [14]. However, most of these analytical methods have some major drawbacks.

HPLC method has been widely developed for screening plant samples and animal tissues containing β-ODAP [15]. In most cases, pre-derivatization of analytes with reagents is a prerequirement for efficient HPLC detection and separation. There are several pre-column derivatization reagents for Reverse Phase Liquid Chromatography amino acid analysis like phenylisothiocyanate (PITC), 6-aminoquinolyl-N-hydroxysccinimidyl carbamate (AQC), 5-dimethylamino-1-naphthalenesulphonyl-chloride (Dansyl), 9-fluorenylmethyl chloroformate (FMOC), 1-fluoro-2,4-dinitrobenzene (FDNB),o-phtalaldehyde (OPA), etc. Extensive derivatization reaction time has been reported for PITC (20 min), AQC (30min), Dansyl (35–50 min), and FMOC (one hour). PITC derivatization involves intricate sample preparation and is sensitive to light. Dansyl derivatization results in uneven production of derivatives. FNDB is toxic which requires careful handling and protective apparel for user [16]. FMOC method requires removal of excess reaction solution with hexane-ethyl acetate which may interfere with the separation of the amino acid derivatives. OPA, PITC derivatization may lead to rapid RP-LC column deterioration [17]. Also, in some cases excess reagent needs to be evaporated making it time consuming process [18]. Further optimization steps for derivatization such as time, extraction solvent and temperature of reaction needs to be studied. These drawbacks make the above derivatization methods time consuming and may result in erratic results. Thus, there is need for an instantaneous and precise method for estimation of β-ODAP.

The other analytical techniques like calorimetric assay is unable to precisely analyse the presence of β-ODAP due to interference of pigments present in plant tissues [19]. Also the nontoxic α-ODAP is determined along with toxic β-ODAP present in plant tissue [20]. Flow injection assay for analysis of neurotoxin β-ODAP, in which immobilized glutamate oxidase in presence of the α-isomer oxidizes the β-isomer [21]. For analysis of food samples matrix associated quenching phenomena is observed by Pati et al. resulting in decrease in sensitivity of this device [22]. As mentioned by Williams, for near infrared reflectance spectroscopy analysis in plants, sample preparation may contribute to 60–70% of errors causing inaccurate measurements [23]. Capillary zone electrophoresis has the disadvantage of a higher operation pH (9.2), a condition that may result in β-ODAP hydrolysis to L-α,β-diaminopropionic acid[24].

To overcome these problems in this paper we propose two techniques: HPLC (without derivatization) and TLC for analysis of anti-nutritive factor. These are simple and effective technique devoid of complex automation. For HPLC analysis various parameters for separation of target compound were optimized. The developed method was validated for linearity, precision, and recovery, limit of detection (LOD) and limit of quantification (LOQ). Thin Layer Chromatography (TLC) was chosen as it is an essential tool for analysis [25]. It has been reported by Tarade et.al that of all the methods reported, for β-ODAP analysis, TLC is simplest, accurate and economical [14]. Unlike other methods mentioned above it, does not require the analyte to have any special properties like UV activity, paramagnetic properties or volatility. The main advantages of TLC are that large number of sample can be separated at one time, instant visualization and low solvent consumption [26] Method was developed for TLC analysis, limit of detection was determined for β-ODAP and substrate of β-ODAP Further, the optimized methods: HPLC and TLC were used to evaluate the bioconversion of β-ODAP from its substrate L-1,2-diaminopropionic acid (L-1,2-DAPA), in presence of seed extract containing the biocatalyst. The amount of β-ODAP formed with time was monitored and quantified.

## Materials and Methods

### Reagents and Chemicals

Standard β-N-oxalyl-2,3-diaminopropionic acid was supplied by Dr S.L.N. Rao from Lathyrus Technologies, Hyderabad, India. L-1, 2-diaminopropionic acid (L-1, 2-DAPA) was a gift from Advanced Laboratory for Plant Genetic Engineering, Indian Institute of Technology, Kharagpur, India. Analytical grade n-Butanol, Acetic acid, Glycerol, Ethyl acetate, Formic acid from Fischer Scientific, MA, USA and HPLC grade Acetonitrile, Isopropanol, Methanol from Spectrochem, Mumbai, India were used. All solutions were prepared with water purified with a Milli-Q system.

### Cultivation of *L*. *sativus* L. plant

Green seeds of matured *Lathyrus sativus* L. plant was chosen. The plant was maintained in garden during winter season in well irrigated soil and for summers in tissue culture laboratory. In the laboratory plants was maintained using standard tissue culture media with some minor modification.

#### Seed sterilization and germination

Seeds of Grass pea (*Lathyrus sativus* L.) low toxin and high yielding line, Nirmal (B1) were chosen as the experimental plant material [27]. Seeds were surface sterilized with Tween-20 for 15 min followed by washing 5–6 times with sterile distilled water and thereafter, exposed to 0.2% HgCl_2_ (Mercuric chloride) for 5 min. Finally, seeds were washed 5–6 times with sterile autoclaved water. Sterilized seeds were germinated in the dark at 25°C overnight on half-strength hormone free MS salts with vitamins and 1% sucrose as mentioned by Murashige and Skoog [28]. Seeds were thereafter maintained at 25°C with 16 hrs light/8 hrs dark condition in a growth chamber.

#### Growth media for plant regeneration and shoot development

Three days old apical meristematic region was isolated from *in-vitro* seedling grown on MSRM medium (MS macro+ micro salts + sucrose1.5%) for multiple shoot regeneration. MS media was used as growth media for plant regeneration and multiple shoots formation. Plantlets with roots were transferred to proliferation medium for hardening by growth in ½ soilrite and ½ soil (autoclaved) for 4 weeks. Finally, plants were transferred to glasshouse for further maturity up to seed harvest.

#### Preparation of seed extract

The extraction of total protein and small molecules was carried out following the manufacturer’s instruction of the P-PER^®^ Plant Protein Extraction Kit, Thermo Scientific. The matured seeds were collected from plant grown in green house. The collected samples were treated with liquid nitrogen to make powder and suspended with protein extraction buffer (25mM Tris-HCl pH 7.5; 500mM NaCl; 10% Glycerol; 1mM EDTA; 1mM PMSF; 0.5% Tween-20; 1mM β-ME). The suspended solutions were homogenized and sonicated on ice. The suspension was centrifuged to sediment the debris other than protein and aqueous layer (total protein) was collected and quantified with protein estimation method [29]

### HPLC

High Performance liquid Chromatography (HPLC) was performed with a Jasco modular LC system equipped with a Rheodyne Injector, binary pump, column oven, auto sampler and thermo controller (set at 4°C) and detection was done using photodiode array detector. All these were controlled by Lab solution LC software (Shimadzu, Japan).

The needle was washed with acetonitrile after each injection to prevent residual samples from previous run. Injection volume was 10μl, flow rate was 0.3ml/min and column temperature was set at 26°C. Sample was applied on reverse phase column, Zorbax Eclipse plus C18 column (4.6X 250mm, 5 μm). Mobile phase A contained water and 0.1% Formic Acid. Mobile phase B was Acetonitrile containing 0.1% Formic Acid. Gradient programme of 15% at 0 min, 15% at 4min, 27% at 7 min, 30% at 10min, 35% at 13 min, 35% at 15 min, 45% at 18min, 100% at 20min, 15% at 25 min was done with flow rate of 0.3 ml/min. Chromatograms were analysed at 254nm.

Working solutions were prepared by mixing stock solution with water to the desired concentration. Fresh stock solution of β-ODAP was prepared before every run. To 1000μg of β-ODAP, 60μl of 0.5M NaHCO_3_ was added and made up to 1ml with water. They were passed through 0.22μm PVDF filter prior to HPLC analysis.

### Validation of the method

The HPLC method has been validated for determination of β-ODAP based on: linearity range, precision (within-day precision and between-day variability), and limit of detection and limit of quantification and recovery

#### Linearity

Linearity was analysed in order to establish the relationship of response (peak area) to β-ODAP concentration. External standard method was used to establish the linearity of the calibration curve for the neurotoxin. Calibration curves were made by plotting peak area versus concentration in the range of 2, 4, 6, 8, 10, 25, 50, 75 and 100μg/ml (n = 9). The linear regression equation (y = mx+c) was used to evaluate linearity. The HPLC analysis was performed in triplicates for all the samples.

#### Limit of detection and quantification

The limit of detection (LOD) for standard β-ODAP was calculated from the calibration curve of the standard β-ODAP. The limit of detection (LOD) and limit of quantification (LOQ) were calculated using the equations: LOD = 3.3 σ/S and LOQ = 10 σ/S where σ represents Standard deviation of response and S represents the slope of the calibration curve.

#### Precision

Precision of the instrument were assessed in one day (within-day precision) and on three days (between-day precision) for retention time and peak area. Precision is calculated by standard deviation (SD) and relative standard deviation (RSD) which is (SD/mean) x100. It was evaluated by analysing three replicates of standard β-ODAP at low, medium and high concentrations of 2, 10 and 100μg/ml.

Recovery was calculated by spiking seed extract with known amount of analyte. It was determined for three concentration 2, 10 and 100 μg/ml in triplicates. % Recovery was calculated as (Recovered conc. /Injected conc.) x 100. The recovered concentration was calculated from equation obtained from standard curve.

### Enzymatic oxalylation of L-1,2-DAPA to β-ODAP

The seed extract was centrifuged at 14.5 x 10^3^ rpm for 30min and clear supernatant was applied on Strata-X RPC Cartridges. Column was conditioned with 1ml of Methanol followed by equilibration in 1ml of water. 500μl of seed extract was loaded on cartridges and then it was washed and eluted twice.

To 100μl of eluent, 50μg of L-1,2-DAPA was added. Reaction was carried out at 22°C and 2μl of samples were collected at 0,1,2,3,4,5,6 and 10 minutes and made up to 20μl. For HPLC analysis the reactions were stopped by adding 50% acetic acid to denature the enzyme.

### Thin Layer Chromatography

Thin layer chromatography was done on Silicagel-60 coated on aluminium foil (11cm X 10cm). TLC protocol with some modification was performed according to Addis and Narayan [2]. TLC jar was saturated with filter paper for 15min to aid equilibration.1mg of β- ODAP was dissolved in 60μl of 0.5M NaHCO_3_ and 940μl MilliQ water. One mg of L-1, 2-DAPA was made up to 1ml with MilliQ water. Spots were applied at a distance of 6cm from the base of plate. A minimum distance of 1.5cm was kept between each spots. Ethyl acetate: acetic acid: formic acid: water 9:3:1:4 were used as mobile phase. Plate was run for 30min and dried on with hair dryer followed by spraying of Ninhydrin reagent. The plates were heated on hot plate maintained at 105°C for 10min. The profile on plate was captured using camera. The R_f_ values of β-ODAP and L-1, 2-DAPA standard were calculated and spot color was noted for identification. The retention of a solute in TLC is characterised by Rf value, which is the distance from the origin to the centre of the separated zone divided by the distance from the origin to the solvent front.

#### Sample preparation

Stock solution of 1000μg/ml of β-ODAP were prepared and spots of 0.6, 1.2, 2.4, 4.8,6 and 8μg/ml were applied to TLC plate.L-1,2-DAPA stock of1000μg/ml was made and spots of 2, 5, 10,20,25,50 μg/μl were applied on a separate TLC plate. Triplicate plates for both ranges were developed and six-point calibration plots were prepared to check the linearity of calibration curve. To determine the lowest concentration of β-ODAP that can be observed on plate, concentrations ranging from 0.2, 0.4 and 0.6μg/ml was applied on plate. Similarly for L-1,2-DAPA 0.5, 1, 2μg/ml were applied, developed and visualized.

The shape of the spot for β-ODAP was circular so Spot area for β-ODAP was quantified by applying the formula for circle i.e. πr^2^. Similarly L-1,2-DAPA spots were elliptical in shape hence spot area was quantified by applying the formula for ellipse i.e. π (r_1_ r_2_)^2^.

#### Application: Bioconversion of L-1,2-DAPA into β-ODAP

10μl of seed extract were suspended in 90μl of Isopropanol: water, 1:9 and kept overnight. They were clarified by centrifugation at 15,000rpm for 30min and supernatant was collected for analysis. To 100μl of clear supernatant, 50μg of L-1,2-DAPA was added. Reaction was carried out at 22°C and 2μl of samples were collected at 0,1,2,3,4,5,6 and 10 minutes and immediately spotted on TLC plate and run was started.

## Result and Discussion

### Optimization of HPLC

Important factors involved in chromatographic separation for retention and resolution of target compound are mobile phase conditions, additive and pH which were optimized in this study. Solvents like acetonitrile and methanol were investigated for separation. Methanol did not show good separation. Acetonitrile gave sharp peak shape hence it was selected. The other benefits of solvent are its low UV-transmittance and low viscosity. In the above mobile phase different additives (0.1% Formic acid and 0.1% DMF) were analysed. The former was favoured as additive since it readily dissolves in acetonitrile and provides sufficient pH control of the mobile phase. Two pH conditions of mobile phase were studied: 2.0 and 5.0 and it was seen that at pH 2.0 the retention time of β-ODAP was 7.4 min as compared to 8.7 min. pH 2.0 made the process faster. 0.3ml/min flow rate was found to be ideal for separation of analyte. We had also used C_18_ Phenomenex Luna column during method development and same retention time was observed.

Gradient elution was performed as sharp peaks are obtained within short separation time [30]. The standard β-ODAP was not chemically modified and resolved into peak with retention time of about 7.4 min. R.Thippeswamy et al. has reported β-ODAP derivatization by OPA with 13.5 min as retention time [13]. The method developed in this paper is faster as no additional step is required for sample modification. In addition to this, HPLC with binary pumps is sufficient for analysis as compared to approach described by Chen et al. where HPLC with ternary pumps are required along with AQC derivatization [20].

### Validation of HPLC method

#### Linearity

**Fig 1:**
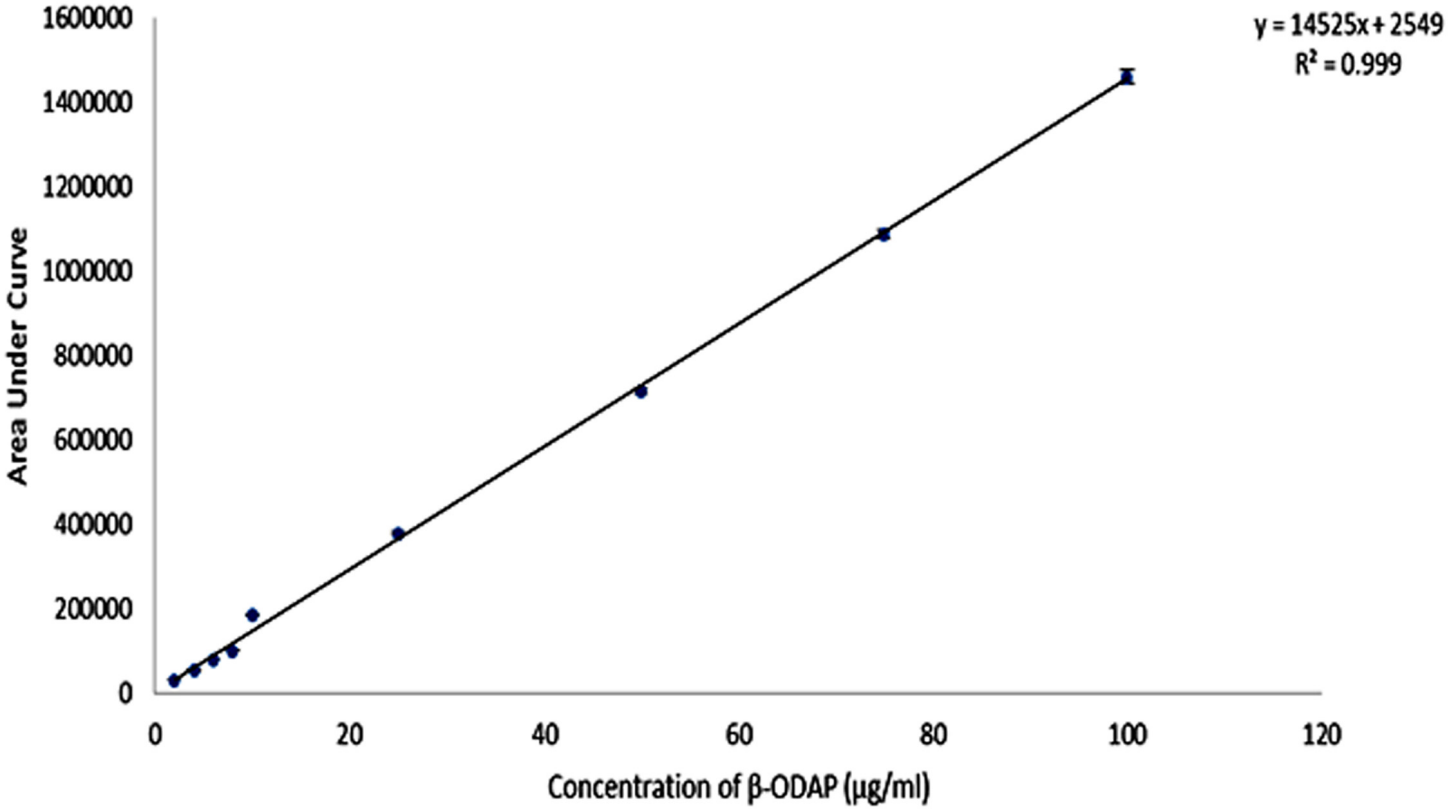
Standard curve for β-ODAP determined by HPLC. AUC (Area Under Curve).

**Fig 2:**
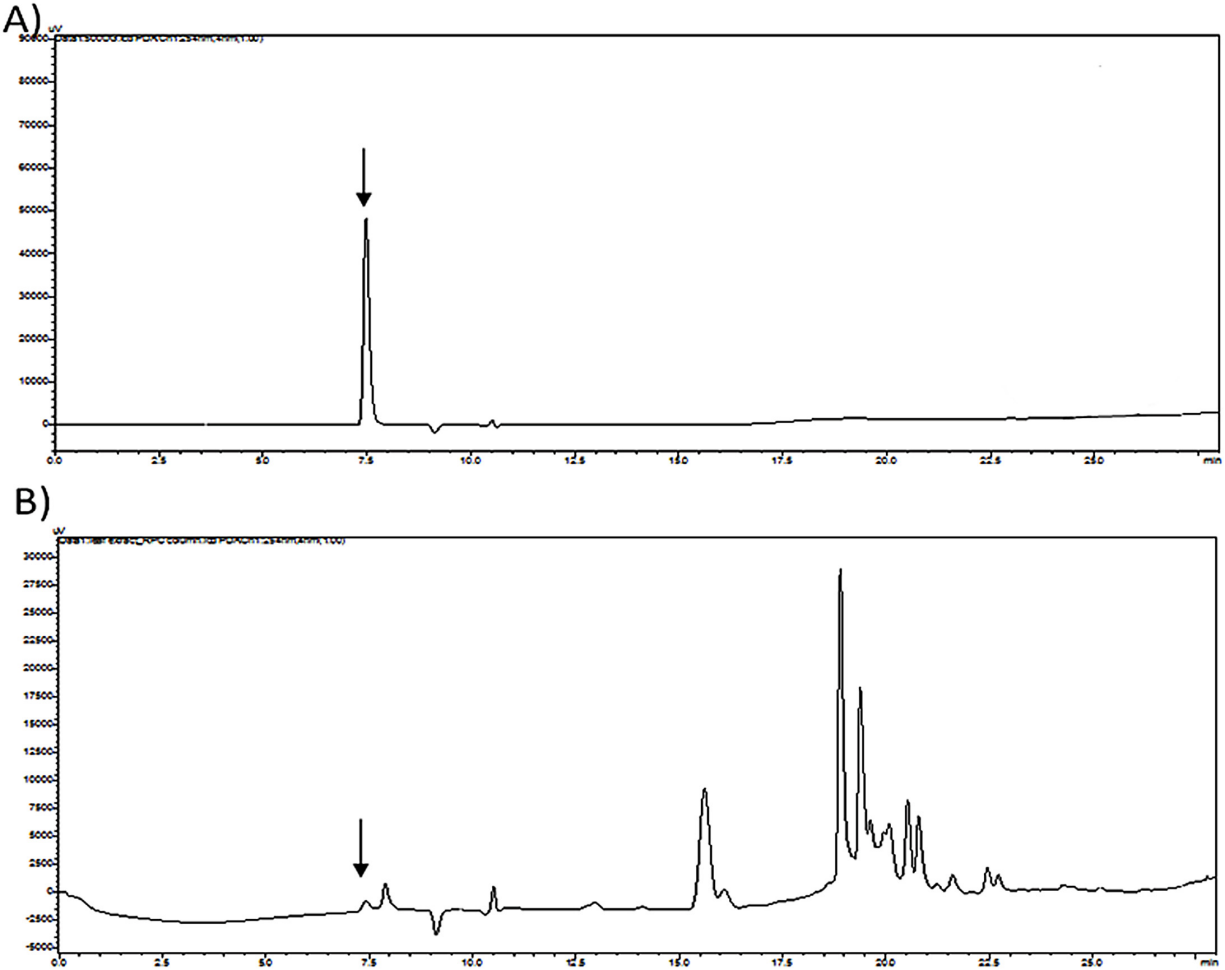
HPLC chromatograms of standard and seed extract of *Lathyrus sativus* L. (A) Standard β-ODAP retention time 7.4 min. Underivatized β-ODAP is indicated by arrow (B) HPLC profile of seed extract having endogenous β-ODAP at 7.4 min (retention time).

With the optimized HPLC conditions, the calibration curve was plotted using 2, 4, 6, 8, 10,25,50,75 and 100 μg/ml concentration of β-ODAP. The calibration curve showed good linearity. The calibration curve for β-ODAP was found to be linear in the concentration range of 2 to 100μg/ml with regression value of 0.99 (Figs 1 and 2A). The equation derived from three replicates was y = 14525x+2549.

#### Limit of detection and quantification

The (LOD) and (LOQ) were determined from the calibration curve of the standard β-ODAP. The LOD and LOQ was determined to be 5.56μg/ml and 16.86μg/ml, respectively.

#### Precision

The intra as well as inter day precision levels for the developed method was analysed in triplicates. The results are presented in Table 1. Three different concentrations were analysed (2, 10 and 100μg/ml). Relative standard deviation (RSD) for Intra-day and inter-day precision was less than 5% signifying good precision of the method so developed (Table 1).

**Table 1:** Intra and Inter-day precision of β-ODAP by HPLC.

Concentration of β-ODAP(μg/ml)	Intraday (RSD%)	Interday (RSD*%)
2	2.79	2.63
10	2.82	3.67
100	1.68	1.13

*RSD is the Relative standard deviation for each sample (n = 3)

The quantitative recoveries of β-ODAP in extract achieved ranged from 92.65%to 106.09% as indicated in Table 2.

**Table 2:** Results for recovery for β-ODAP.

Concentration of β-ODAP(μg/ml)	Found concentration	%Recovery
2	1.85	92.65
4	4.23	105.76
6	5.69	94.80
8	7.63	95.39
10	10.61	106.09
100	99.98	99.97

### Biosynthesis and quantification of product formed

The sample preparation was simple involving concentration of β -ODAP and removal of bulk contaminants from the extract. Solid phase extraction was done, which involve use of functionalized polymeric sorbent for removal of unwanted compounds on the basis of chemical properties. After extraction HPLC analysis was done directly. Majority of derivatization procedures involve reaction with amino (primary and secondary amines) and carboxyl groups. Free amino acid of proteins and peptides present in extract may undergo modification along with target compound (ODAP) resulting in interference in ODAP measurement. Hence this step was not performed.

The retention time and absorption spectra of the standard was compared with the chromatogram of seed extract. The peak with same retention time as that of standard β-ODAP was identified as the neurotoxin. The equation obtained from calibration curve was used to determine the concentration of the product so formed.

Plant extract being complex mixture of compounds was analysed by HPLC to examine the presence of endogenous β-ODAP along with other biomolecules. The concentration of compound of interest was found to be 0.71 μg/ml, observed at 7.4 which corresponded to retention time of standard β-ODAP (Fig 2). The HPLC run for 25min was sufficient to separate all the compounds present in preparation. The impurities present in extract eluted at 18min without interfering with target compound.

**Fig 3:**
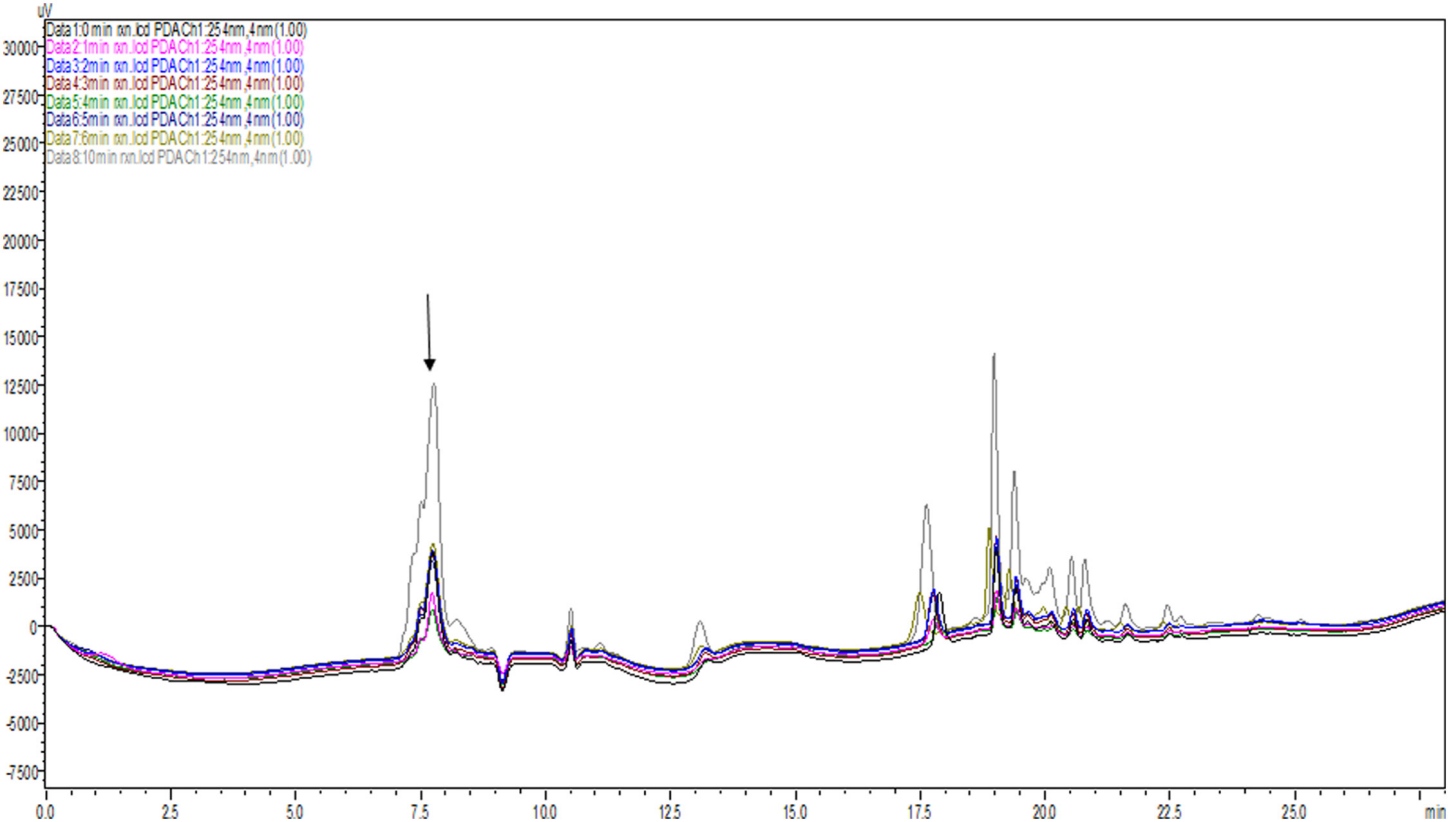
Over layered chromatograms of bioconversion at different time points. Chromatograms of 0,1,2,3,4,5,6 and 10 min were over layered. Product is indicated by an arrow. Reaction mixture was detected at 254 nm by Photo Diode Array (PDA).

The biosynthesis was performed in the presence of seed extract with known concentration of L-1, 2-DAPA. The reaction was monitored for 0 to 6^th^ min at interval of one min and 10th min to evaluate product formation (Fig 3). HPLC chromatograms so obtained were over layered to observe the progress of reaction and by-product peaks formed in reaction. The peak indicated with arrow in Fig 3, obtained after biosynthesis presents two shoulders. These peaks might be the by-product formed during the reaction with similar polar properties as β-ODAP hence elutes immediately before and after the formed β-ODAP. In Fig 2A, chromatogram of standard β-ODAP and seed extract (Fig 2B) is presented where no shoulder is seen confirming the formation of new products. It is observed that with time β-ODAP area under peak increase with time.

The product formation was quantified and endogenous β-ODAP was deducted to quantify the actual neurotoxin formation. Tenth minute sample exhibited 10.85μg of β-ODAP (Table 3). Further monitoring was done at 30^th^ minute which showed decrease in the amount of neurotoxin synthesized (results not shown).

**Table 3:** Quantification of β-ODAP formed with time.

Time (min)	β-ODAP formed(μg)
0	0.62
1	2.64
2	2.93
3	5.8
4	6.15
5	6.39
6	6.56
10	10.85

There are two proposed steps for biosynthesis of β-ODAP by Malathi et al. [31]. Two enzymes: oxalyl CoA synthetase and β-ODAP synthease are involved in biosynthesis of β-ODAP. The first reaction is catalyzed by oxalyl-CoA synthetase in which substrates oxalate and coenzyme A, is converted to Oxalyl-CoA in presence of ATP [Eq 1]. The second reaction is catalysed by β-ODAP synthease, which is specific to *Lathyrus sativus* L. and acts on the product of first step Oxalyl-CoA and L-1,2-diaminopropionic acid to form the product β-ODAP and by product CoA [Eq 2]. Oxalyl-CoA, is the donor for the oxalyl moiety during *in-vitro* enzymatic synthesis of β-ODAP. This biotransformation reaction might be employed to detect the presence of biocatalyst responsible for synthesis of the neurotoxin.

$$\begin{array}{l} \;\\ \text{Oxalate }+\text{ATP }+\text{ Coenzyme}-\text{A}\overset{{\text{Mg}}^{2+}}{\underset{\text{Oxalyl}-\text{CoA synthetase}}{⇄}}\text{Oxalyl}-\text{CoA }+\text{ AMP }+\text{PPi }\\ \; \end{array}$$ (1)

$$\begin{array}{l} \text{Oxalyl}-\text{CoA + L-1,2-diaminopropionic acid }\underset{\beta -\text{ODAP synthease}}{\to }\;\beta -\text{ODAP }+\text{ CoA}\\ \text{ ​ }\;\; \end{array}$$ (2)

Product formation indicated presence of active and stable enzyme in sample. There was no requirement to supplement the extract with any stabilizers. Sample preparation conditions and buffer components used for solid phase extraction were not detrimental for enzyme. The method developed can be employed to determine a minimum concentration of 16.86 μg/ml (LOQ) of β-ODAP in plant extract with accuracy and precision. This method can be used for low toxin variety like P28, P 94–3 and P90-2 as these varieties are reported to contain less than ~0.1% of β- ODAP [32].

### Thin layer chromatography

#### Calibration and useful detection range for β-ODAP

Chromatography involves the selective separation based on the adsorption of analyte on the stationary solid phase and desorption by mobile solvent phase. Hence to achieve selectivity three different compositions of TLC mobile phase were investigated during method development. Toluene: ethyl acetate: formic acid (5:4:1), ethylacetate: acetic acid: formic acid: water (9:3:1:4) and butanol: acetic acid: water (4:1:1) contained polar solvents of increasing strength as per the Elutrophic series [33]. Butanol: acetic acid: water showed the best separation as the compounds were highly soluble and migrated fastest on plate. The above screened solvents were used for determining the relative mobility of standard β-ODAP and L-1, 2-DAPA.

**Fig 4:**
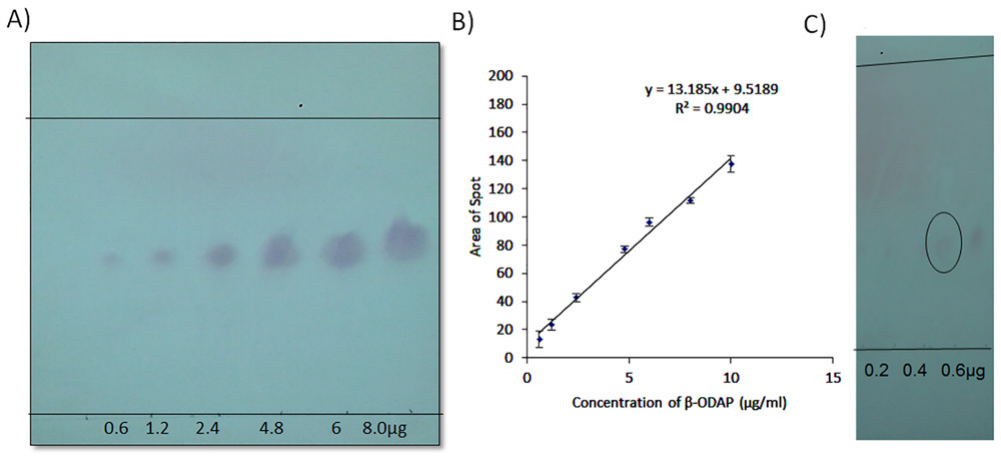
Results for β-ODAP detection by TLC. A) Visualization of standard β-ODAP on TLC plate. B) Calibration curve for β-ODAP C) Determination of Limit of detection for β-ODAP. The lowest concentration 0.4μg visible is circled.

The relative mobility of β-ODAP was determined to be 0.37 (Fig 4A). Calibration plot showed linear relationship between the concentrations of β-ODAP applied to the spot area obtained. A seven point calibration graph was plotted with r^2^ value obtained was greater than 0.97 (Fig 4B). In order to determine the lowest detection range, 0.2, 0.4 and 0.6μg of β-ODAP were applied. As seen in Fig 4C, this method enabled visualization of compound as low as 0.6μg making it a sensitive method. β-ODAP develops as circular shape and L-1,2-DAPAas oval shape which was seen after ninhydrin staining.

The method developed here with mobile phase, ethyl acetate: acetic acid: formic acid: water 9:3:1:4 enabled separation in 30min as compared to 2.5hr as mentioned by Paradkar et al. for detection by TLC method [34].

#### Calibration and useful detection range for L-1, 2-DAPA

**Fig 5:**
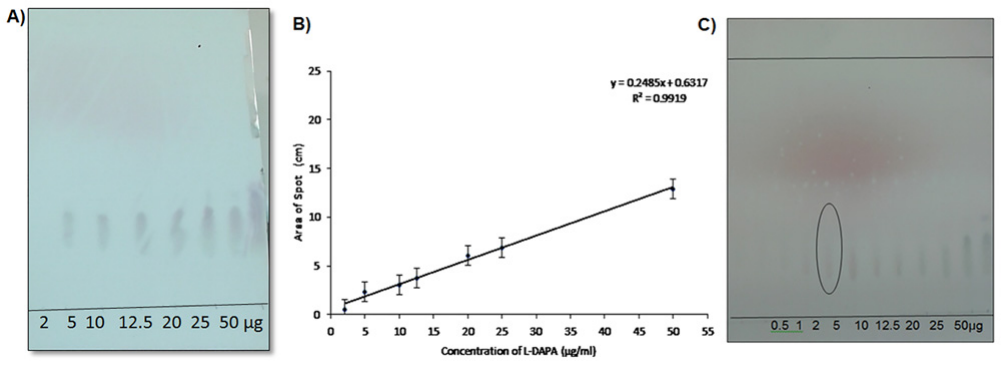
Results for L-1, 2-DAPA detection by TLC. **A)** Visualization of standard L-1,2-DAPA on TLC plate B) Calibration curve for L-1,2-DAPA C) Determination of Limit of detection for L-1,2-DAPA. The lowest concentration visible is circled.

The relative mobility (R_f_) of L-1, 2-DAPA was determined to be 0.26 (Fig 5A). Calibration curve was plotted for seven different concentrations and r^2^ value was determined as 0.99. There was a linear response between concentration of standard of L- DAPA and spot area obtained (Fig 5B) The lowest limit of detection for L-1,2-DAPA was 5μg. Lower concentrations that is 0.5,1 and 2μg/μl could not be clearly visualized on the plate (Fig 5C).

### Biosynthesis of L-1, 2-DAPA to β-ODAP by plant extract

**Fig 6:**
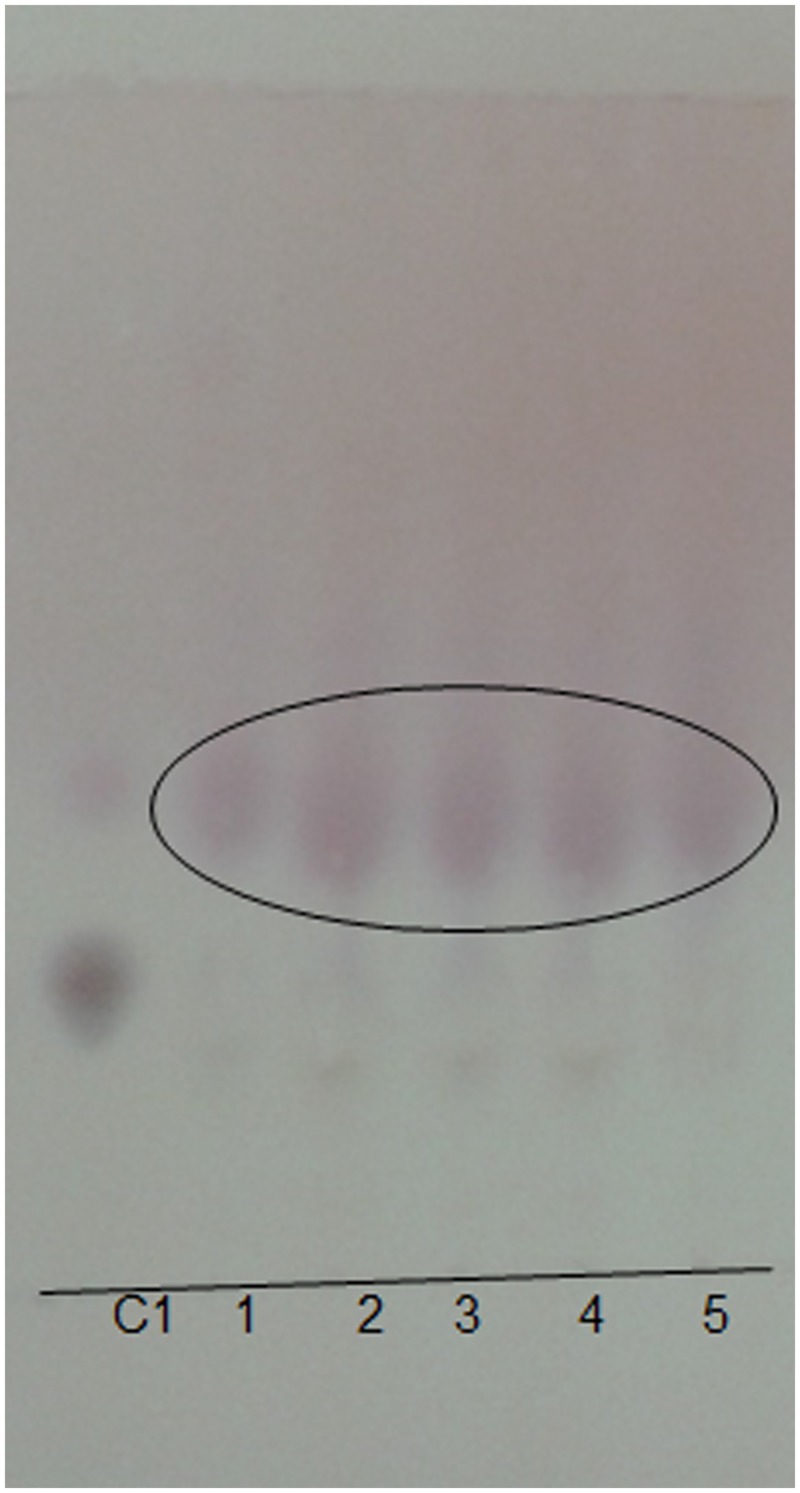
Observation of Bioconversion of L-1, 2-DAPA into β-ODAP catalyzed by seed extract. Enzymatic catalysis of 50μg of L-1, 2-DAPA to β-ODAP at 1min, 2min, 3min, 5min, 6min and 10min. Encircled spots are product synthesized in reaction mixture. C1 is standard for L-1, 2- DAPA.

This TLC based method so developed for detection was investigated for bioconversion of DAPA to β-ODAP in presence of seed extract. The reaction mixture when analysed by TLC showed presence of three resolved bands. As seen in TLC plate (Fig 6) standard L-1,2-DAPA is spotted (C1) and seen with R_f_ value of 0.26 (1^st^ spot). In samples 1–5, a prominent spot with R_f_ value 0.37 which corresponds to standard β-ODAP, were observed. Below β-ODAP, in trace amount the spot observed is unconverted L-1, 2-DAPA which matched to R_f_ value of 0.26.

## Conclusion

In this manuscript, TLC and HPLC analysis methods for β-ODAP were established. HPLC method exhibits good linearity, precision and recovery for detection of underivatized β-ODAP. The advantage of HPLC approach is reduction of preprocessing time as well as reduced loss of target analyte, as no prior derivatization is required. *In-vitro* conversion of L-1,2,-DAPA to β-ODAP indicates the presence of active β-ODAP synthetase and β-ODAP synthase in the crude extract. This biocatalytic reaction for product formation was confirmed and this approach is reported for first time. This assay can be used where Lathyrus strains are developed by knockout of the synthetic pathway of β-ODAP.

The linearity in the range from 2 to 100μg/ml of β-ODAP was found to be suitable for analysis of the toxin produced in Lathyrus strains. Further, this method can be applied for monitoring the presence of grass pea a common adulterant, in expensive legume seeds such as chickpea (*Cicer arietinum*) and red gram (*Cajanus cajan*). Both HPLC and TLC are simple and affordable methods making it viable for detection and quantification of large volume of samples obtained after harvest.
